# Supplementary material for: Unidirectional guided-wave-driven metasurfaces for arbitrary wavefront control
Source: Nat Commun. 2024 Jul 16;15:5992. doi: 10.1038/s41467-024-50287-z (PMC11252367; doi:10.1038/s41467-024-50287-z)
Supplement: Supplementary file 1 — Supplementary Information [file 41467_2024_50287_MOESM1_ESM.pdf]

Supplementary Information for

Unidirectional guided-wave-driven metasurfaces for arbitrary wavefront control

Shiqing Li[1], Kosmas L. Tsakmakidis[2]*, Tao Jiang[3], Qian Shen[1], Hang Zhang[1], Jinhua Yan[1], Shulin Sun[4] and Linfang Shen[1]*

[1]*Department of Applied Physics, Zhejiang University of Technology, Hangzhou 310023, China.*

[2]*Section of Condensed Matter Physics, Department of Physics, National and Kapodistrian University of Athens Panepistimioupolis, Athens GR-157 84, Greece.*

[3] *Yangtze Delta Region Institute (Huzhou), University of Electronic Science and Technology of China, Huzhou 313001, China.*

[4]*Shanghai Engineering Research Centre of Ultra Precision Optical Manufacturing, Department of Optical Science and Engineering, School of Information Science and Technology, Fudan University, Shanghai 200433, China.*

CONTENTS

1. Propagation loss and phase constant of microwave unidirectional mode
2. Phase shift from the coupling between two types of USMPs
3. Beam deflection with a USMP-driven metasurface
4. Proof of the phase inequality
5. Evaluation of efficiency of the proposed metadevices
6. Holographic metasurface for super-resolution imaging
7. The dispersion relation of unidirectional mode in the presence of external magnetic field
8. The generated OAMs with different orders at different frequencies
9. Tuning the output OAMs via external magnetic field
10. Tailoring the output OAMs through structural parameter
11. Tunable ring-cavity OAM source based on the USMP-driven metasurface using an alternative approach
12. Terahertz USMPs with unique dispersion
13. Structural details of the designed USMP-driven metasurfaces for wavefront control
14. Comparison of the proposed USMP-driven metasurface with selected prior art.

1. Propagation loss and phase constant of microwave unidirectional mode

To effectively excite the unidirectional mode, the actual waveguide samples in the experiment consist of three sections: a longer central section that is the microwave unidirectional waveguide, and two short sections at both ends that are rectangular metal waveguides filled with ceramic material. We fabricated two waveguide samples, with the unidirectional waveguide having a length of L_1 = 20 mm in sample 1 and a length of L_2 = 40 mm in sample 2. The other parameters of the two samples are identical. Although the waveguide samples are not uniform waveguide structures, we can still evaluate the propagation loss and phase constant of the unidirectional mode from the S21 parameters of the two waveguide samples. The propagation length of the unidirectional mode can be evaluated from the magnitudes of the S21 parameters, given by $L_p = \ln[|S21(1)|/|S21(2)|]/(L_2–L_1)$, where S21(1) and S21(2) are the S21 parameters of samples 1 and 2 respectively. The obtained results are shown in Fig. S1c. At the center (2.69 GHz) of the unidirectional frequency window, the propagation length is about $8\lambda_0$. The phase constant of the unidirectional mode can be evaluated from the phases of the S21 parameters, given by $k = \{Arg[S21(2)] - Arg[S21(1)]\}/(L_2–L_1)$, and the obtained results are shown in Fig. S1d.

Using the finite-element method, we numerically simulated the transmission of waves in the waveguide sample 2. Figure S1b presents the simulated magnetic field intensity at the frequency 2.69 GHz. It is observed that the output power from the unidirectional waveguide partially excites a unidirectional surface wave at the YIG-ceramic interface. This surface wave is finally captured by the bottom metal and absorbed by the YIG material, resulting in a smaller S21. By numerically analyzing the dependence of the amplitude and phase of the field on the propagation distance in the unidirectional waveguide, we can determine the real (k) and imaginary parts (α) of the propagation constant. The propagation length of the unidirectional mode equals to $L_p = 1/(2\alpha)$. The propagation length and dispersion relation of the unidirectional mode obtained from numerical simulations are also shown in Figs. S1c and S1d. In our unidirectional waveguide, the field of the unidirectional mode is uniformly distributed in the lateral direction, hence it is physically equivalent to that in the (2D) metal-air-

YIG layered structure, which is described by the dispersion equation (2). For the actual YIG with loss ($\Delta H = 5$ Oe), we numerically solved the dispersion equation (2), and the calculated results are also plotted in Figs. S1c and S1d. It can be seen that the results for both the propagation length and the dispersion relation of the unidirectional mode are in good agreement among the experiments, simulations, and theory.

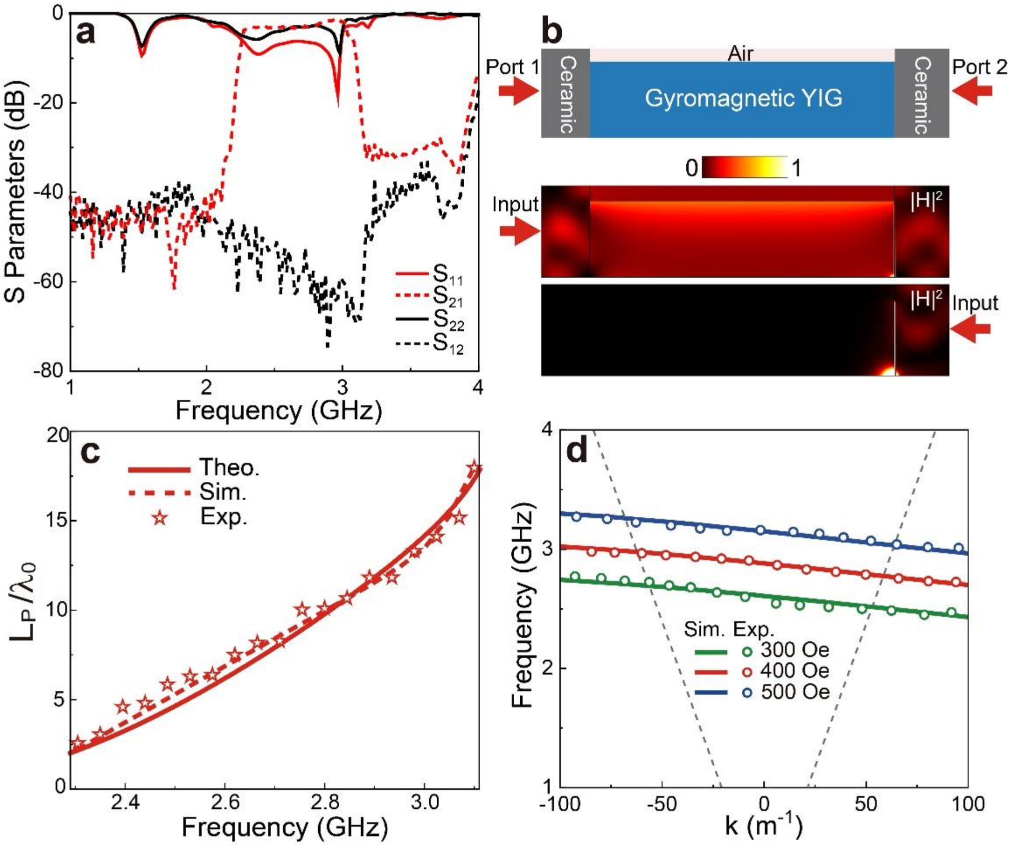

Fig. S1. Experimental measurement and numerical simulation of the microwave unidirectional waveguide. a The measured S-parameters of the waveguide sample 2. **b** Simulated magnetic-field intensity ($|H|^2$) distributions at 2.69 GHz for forward and reverse transmissions. Top: a schematic diagram of the waveguide sample. **c** Theoretical (solid line), simulated (dashed line), and experimentally measured (star) propagation length as a function of frequency. **d** Simulated (solid lines) and measured (circles) dispersion relations of the unidirectional waveguide for external magnetic fields $H_0 = 300$, 400, and 500 Oe.

2. Phase shift from the coupling between two types of USMPs

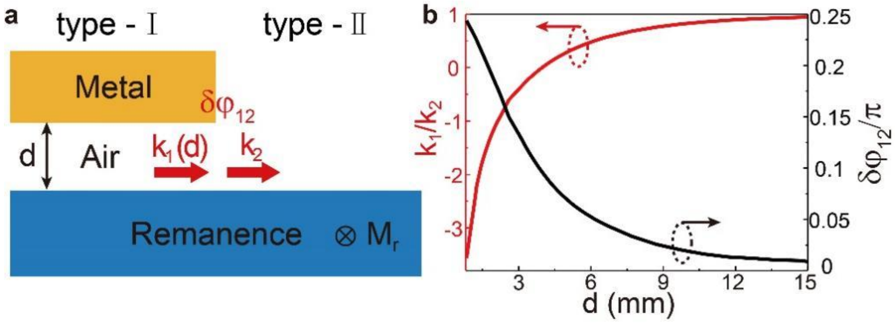

Fig. S2. Phase shift from the coupling between two USMPs. a Schematic diagram of the phase shift $\delta\varphi_{12}$ from the coupling between two different USMPs. **b** Simulated $\delta\varphi_{12}$ and matching degree (indicated by k_1/k_2) between the two USMPs with the variation of d. The phase shift $\delta\varphi_{12}$ increases rapidly with the mismatch between the two USMPs, and is far smaller than π in general.

3. Beam deflection with a USMP-driven metasurface

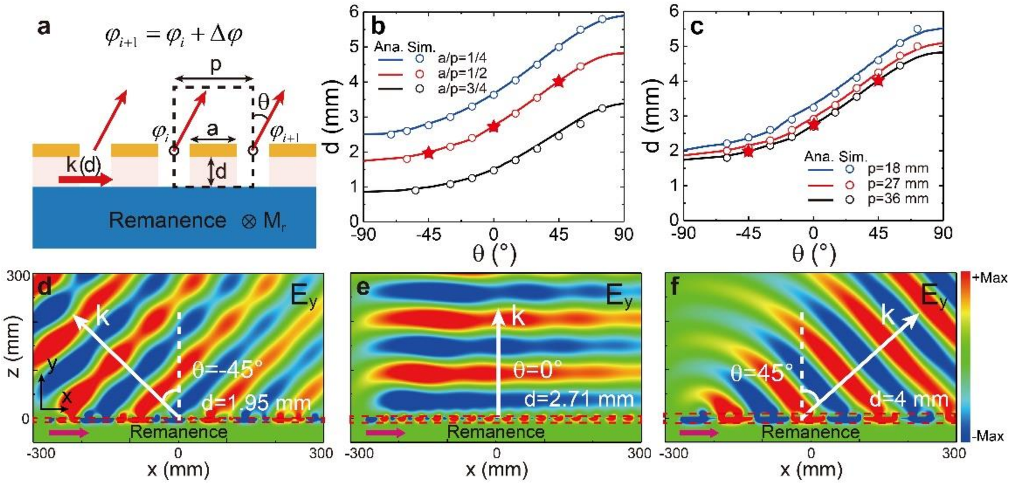

Fig. S3. Beam deflection with the metasurface. a Schematic diagram of the uniform metasurface formed by alternating type-I and type-II waveguides. **b**, **c** The analytic [solid lines, obtained by $\theta = \sin^{-1}(k_x/k_0)$] and numerical (circles) results of the radiation angle θ as a function of d. The period length is fixed at p = 36 mm in (b), and duty ratio is fixed at a/p = 1/2 in (c). **d-f** Simulated E_y field patterns radiated from the uniform metasurfaces for (d) d = 1.95 mm, (e) d = 2.71 mm, and (f) d = 4, which are also marked by red stars in (b) and (c). In the simulations, the excited source is placed in the air layer at the left side of the uniform metasurfaces.

4. Proof of the phase inequality

In order to verify the inequality

$$-k_0 p \leq \arg\{U_0(x+p)\} - \arg\{U_0(x)\} \leq k_0 p, \tag{S1}$$

with $U_0(x) = \int_{-k_0}^{k_0} A(k_x)\exp(jk_x x)dk_x$, it is necessary to first prove the following basic inequality

$$\Delta\phi_{\min} \leq \left|\arg\left(a_1 e^{i\Delta\phi_1} + a_2 e^{i\Delta\phi_2}\right) - \arg(a_1 + a_2)\right| \leq \Delta\phi_{\max}, \tag{S2}$$

where $\Delta\phi_{\min} = \min(|\Delta\phi_1|, |\Delta\phi_2|)$, and $\Delta\phi_{\max} = \max(|\Delta\phi_1|, |\Delta\phi_2|)$. In the proof, there are three different cases to consider: (1) $\Delta\phi_1 \geq \Delta\phi_2 \geq 0$, (2) $\Delta\phi_1 \leq \Delta\phi_2 \leq 0$, (3) $\Delta\phi_1 \geq 0$, $\Delta\phi_2 \leq 0$. In Fig. S3, through the graphical representation and operation of complex numbers, the following inequality is graphically obtained for case 1: $\Delta\phi_2 \leq \arg(a_1 e^{i\Delta\phi 1} + a_2 e^{i\Delta\phi 2}) - \arg(a_1 + a_2) \leq \Delta\phi_1$. In a similarly, we can obtain the inequality $\Delta\phi_1 \leq \arg(a_1 e^{i\Delta\phi 1} + a_2 e^{i\Delta\phi 2}) - \arg(a_1 + a_2) \leq \Delta\phi_2$ for case 2 ($\Delta\phi_1 \leq \Delta\phi_2 \leq 0$), and the inequality $\Delta\phi_2 \leq \arg(a_1 e^{i\Delta\phi 1} + a_2 e^{i\Delta\phi 2}) - \arg\ (a_1 + a_2) \leq \Delta\phi_1$ for case 3 ($\Delta\phi_1 \geq 0$, $\Delta\phi_2 \leq 0$). To summarize, we have proved the basic inequality (S2).

In Eq. (S2), only two pairs of complex numbers are included. Obviously, this inequality can be generalized to the general case of arbitrary (N) pairs of complex numbers, and the generalized inequality has the form

$$\Delta\phi_{\min} \leq \left|\arg\left(\sum_{n=1}^{N} a_n e^{i\phi_n}\right) - \arg\left(\sum_{n=1}^{N} a_n\right)\right| \leq \Delta\phi_{\max}, \tag{S3}$$

where $\Delta\phi_{\min} = \min(|\phi_1|, |\phi_2|, \cdots, |\phi_N|)$ and $\phi_{\max} = \max(|\phi_1|, |\phi_2|, \cdots, |\phi_N|)$. In Eq. (S1), the complex physical quantities can also be expressed as $U(x) = \sum^N a_n$ and $U(x+p) = \sum^N a_n e^{ik_n p}$, where $a_n = A(k_n)e^{ik_n x}$ and $k_n \in [-k_0, k_0]$, therefore they satisfy the inequality (S1), i.e., $|\arg\{U_0(x+p)\} - \arg\{U_0(x)\}| \leq k_0 p$.

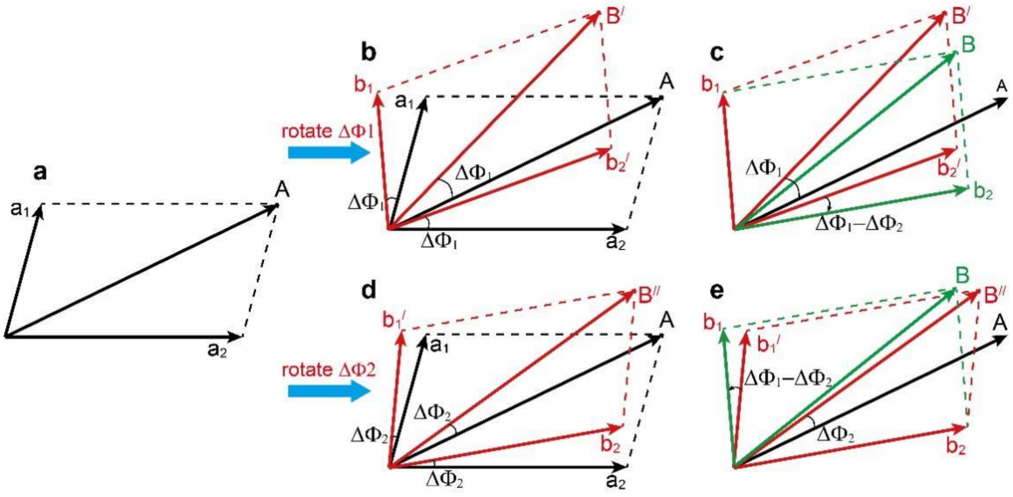

Fig. S4. Graphical representation and operation of complex numbers. We consider four complex numbers a_1, a_2, b_1, and b_2, where $b_1 = a_1e^{i\Delta\phi 1}$ and $b_2 = a_2e^{i\Delta\phi 2}$, and assume $\Delta\phi_1 \geq \Delta\phi_2 \geq 0$. The addition of $A = a_1+a_2$ and $B = b_1+b_2$ is performed graphically on the complex plane. **a** Graphically representing a_1, a_2, and their sum. **b** Rotating a_1, a_2 and A together by an angle $\Delta\phi_1$, we get b_1, b_2' and B', respectively. **c** Dragging down b_2' by $\Delta\phi_1 - \Delta\phi_2$, we then obtain $b_2 = a_2e^{i\Delta\phi 2}$, then add up b_1 and b_2, we finally get B. Obviously, $\arg(B) - \arg(A) \leq \arg(B') - \arg(A)$, this means $\arg(a_1e^{i\Delta\phi 1}+a_2e^{i\Delta\phi 2}) - \arg(a_1+a_2) \leq \Delta\phi_1$. **d** Rotating a_1, a_2 and A by an angle $\Delta\phi_2$, we get b_1', b_2 and B'', respectively. **e** Dragging up b_1' by $\Delta\phi_1$–$\Delta\phi_2$, we then obtain $b_1 = a_1\mathrm{e}^{i\Delta\phi 1}$, then add up b_1 and b_2, we finally get B. Obviously, $\arg(B) - \arg(A) \geq \arg(B'') - \arg(A)$, this means $\arg(a_1e^{i\Delta\phi 1}+a_2e^{i\Delta\phi 2}) - \arg(a_1+a_2) \geq \Delta\phi_2$. To sum up, we verify the inequality $\Delta\phi_2 \leq \arg(a_1e^{i\Delta\phi 1}+a_2e^{i\Delta\phi 2}) - \arg(a_1+a_2) \leq \Delta\phi_1$.

5. Efficiency evaluation of the proposed metadevices

The efficiency of the USMP-driven metasurface is evaluated and discussed in this part. As shown in Fig. S4, we utilized a USMP-driven metasurface to excite 2D Bessel beam as an example, and introduce its efficiency calculation method. The extracted waves of the metasurface have uniform amplitudes and spatial-variant phases as described by $\varphi(x) = -\alpha|x|$. Two efficiency characteristics are considered here, and they are energy efficiency (EE) and utilization efficiency (UE). The energy efficiency is defined by:

$$\mathrm{EE} = \frac{P_{\mathrm{fun}}}{P_{\mathrm{in}}}, \tag{S4}$$

where P_{fun} represents the output power of phase-modulated free-space wave, while P_{in} represents the input power of unidirectional guided mode in the waveguide. P_{in} and P_{fun} are obtained by integrating the energy flux over the regions s_1 and s_3, which were marked with dotted lines in Fig. S4. The calculated EEs of designed meta-device for 2D Bessel beam generation is 57% at the designed frequency 2.69 GHz. Evidently, the energy efficiency can be effectively improved by increasing the length of the USMP-driven metasurface in the *x* direction, or by connecting multiple meta-devices via the unidirectional waveguide.

In order to more rationally evaluate the efficiency of USMP-driven metasurface, we introduced utilization efficiency (UE), which is defined by:

$$\mathrm{UE} = \frac{P_{\mathrm{fun}}}{P_l}, \tag{S5}$$

where P_l represents the total power loss during the propagation of unidirectional guided modes over the metasurface. $P_l = P_{\mathrm{in}} - P_d$, and P_d can be obtained by integrating the energy flux over the region s_2, which is marked with dotted line in Fig. S4. The energy utilization efficiency for the metadevice is 72% at the working frequency, which implies that most of the lost energy of unidirectional modes can be converted to preset free-space functionalities. Only a small part

of the consumed energy is exhausted due to material absorption. Similar numerical calculations have also been done for 3D beam focusing and 3D Bessel beam generation, and the utilization efficiency for these metadevices are presented in the main text.

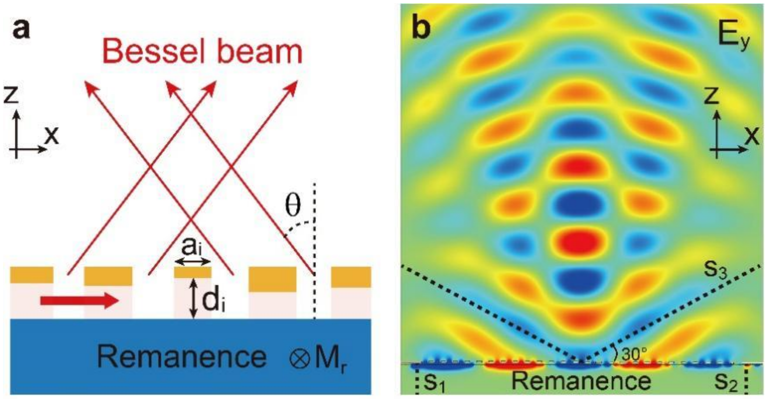

Fig. S5. Efficiency evaluation of the proposed metadevices. a Schematic diagram of the 2D USMP-driven meta-axicon. **b** Simulated Re[E_y] field patterns for Bessel beam generation studied in (a). The integration regions s_1, s_2, s_3, were marked with black dashed lines, are used to calculate the input (P_{in}) and decreased (P_d) powers of unidirectional mode, and the output power (P_{fun}) in free space, respectively.

6. Holographic metasurface for super-resolution imaging

A holographic metasurface is designed for near-field imaging of the Greek letter "ϕ". This metasurface, formed by a 2D array of L-shaped meta-cells with the deep wavelength size $\lambda/20$ (λ is the design wavelength), is expected to produce wavelength-sized ϕ image at the height 0.1λ. For the ideal image of ϕ, the radius of the ring is 0.45λ and the length of the vertical line is 2λ, with a linewidth of 0.2λ for both. It was numerically demonstrated that our metasurface is capable of achieving this super-resolution imaging, as the extracted wavefront contains not only propagating wave components but also evanescent wave components.

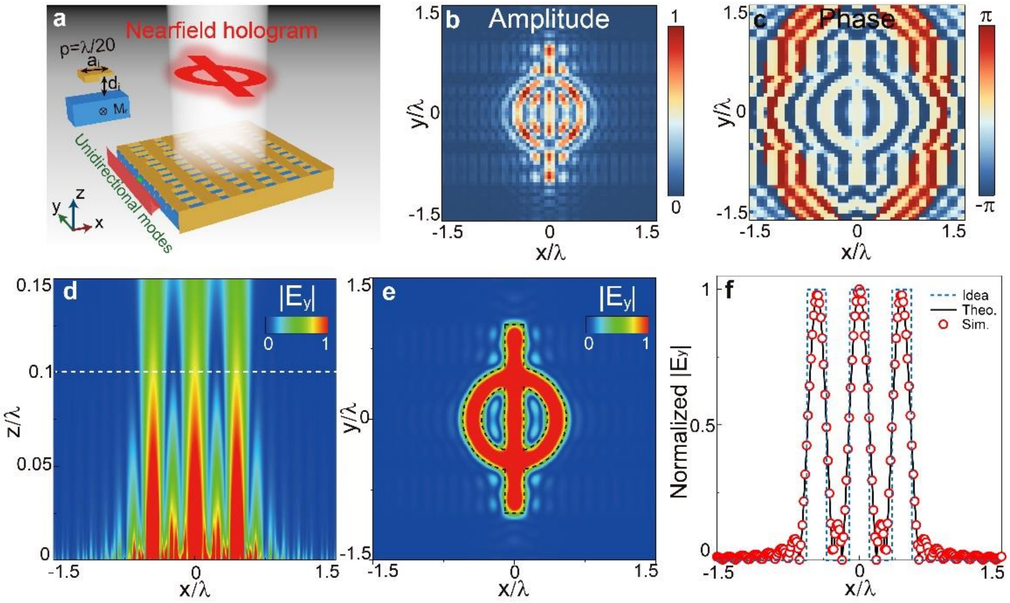

Fig. S6. Demonstration of super-resolution imaging of the Greek letter "ϕ" with a USMP-driven metasurface. a Schematic diagram of the USMP-driven holographic metasurface for near-field imaging. The meta-cells comprising the metasurface have the deep wavelength size $p = \lambda/20$, where λ is the vacuum wavelength for the design frequency 2.69 GHz. **b** Designed amplitude and **c** phase profiles of the wavefront extracted from the metasurface. **d** Simulated $|E_y|$ pattern on a longitudinal slice of $y = 0$. The dashed line marks the location of the image plane at $z = 0.1\lambda$. **e** Simulated $|E_y|$ pattern on the image plane. **f** Distribution of normalized E_y amplitude along the x axis on the image plane, which indicates that the linewidths of the circular ring and straight line are only 0.2λ.

7. The dispersion relation of unidirectional mode in the presence of external magnetic field

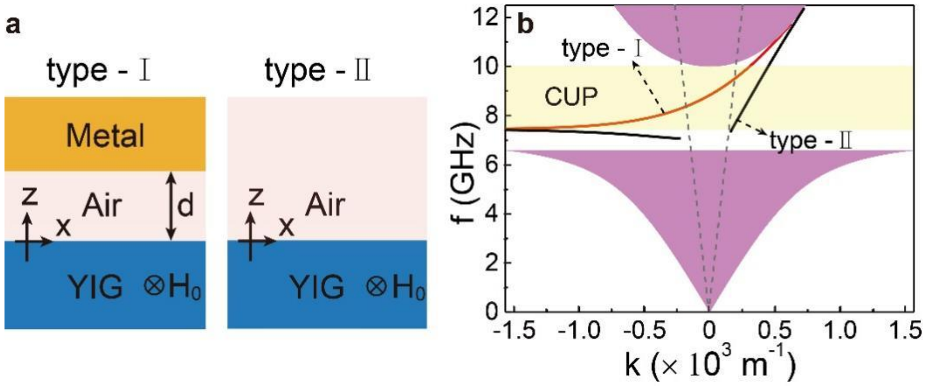

Fig. S7. Two types of USMPs. a Schematic diagrams of waveguides supporting type-I and type-II USMPs in the presence of external magnetic field (H_0). **b** Dispersion diagram of the two waveguides. Here, $\omega_m = 10\pi \times 10^9$ rad/s ($f_m = 5$ GHz), and $H_0 =$ 1785 Gs.

8. The generated OAMs with different orders at different frequencies

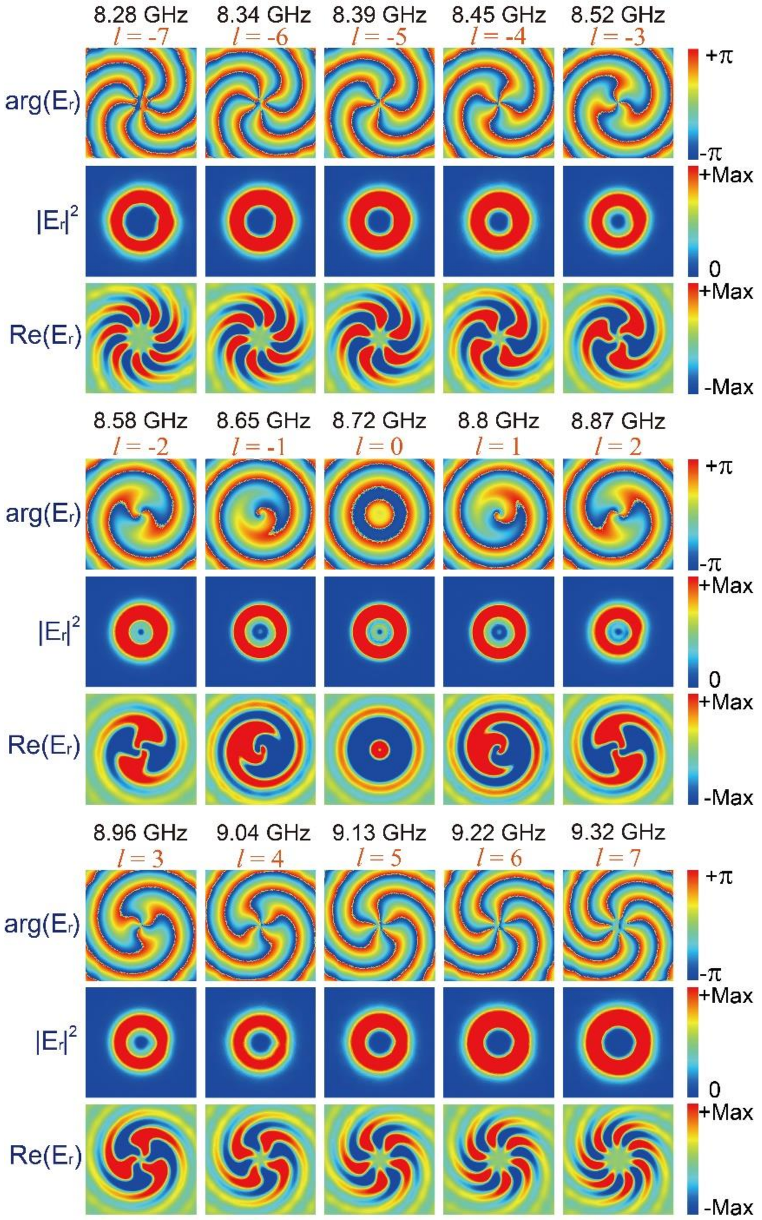

Fig. S8. OAM tuning through frequency. Simulated phase, intensity and real part profiles of radial electric-field components of emitted OAM states at different frequencies. The ring cavity is composed of 43 meta-cells with p = 6 mm and a = 4 mm. The external magnetic field is fixed at H_0 = 1785 Gs. The resulting topological charge of the OAMs radiation can be seen by the number of 2π phase evolution along the circumference, which ranges from −7 to +7 for the considered frequency range [8.28, 9.32] GHz.

9. Tuning the output OAMs via external magnetic field

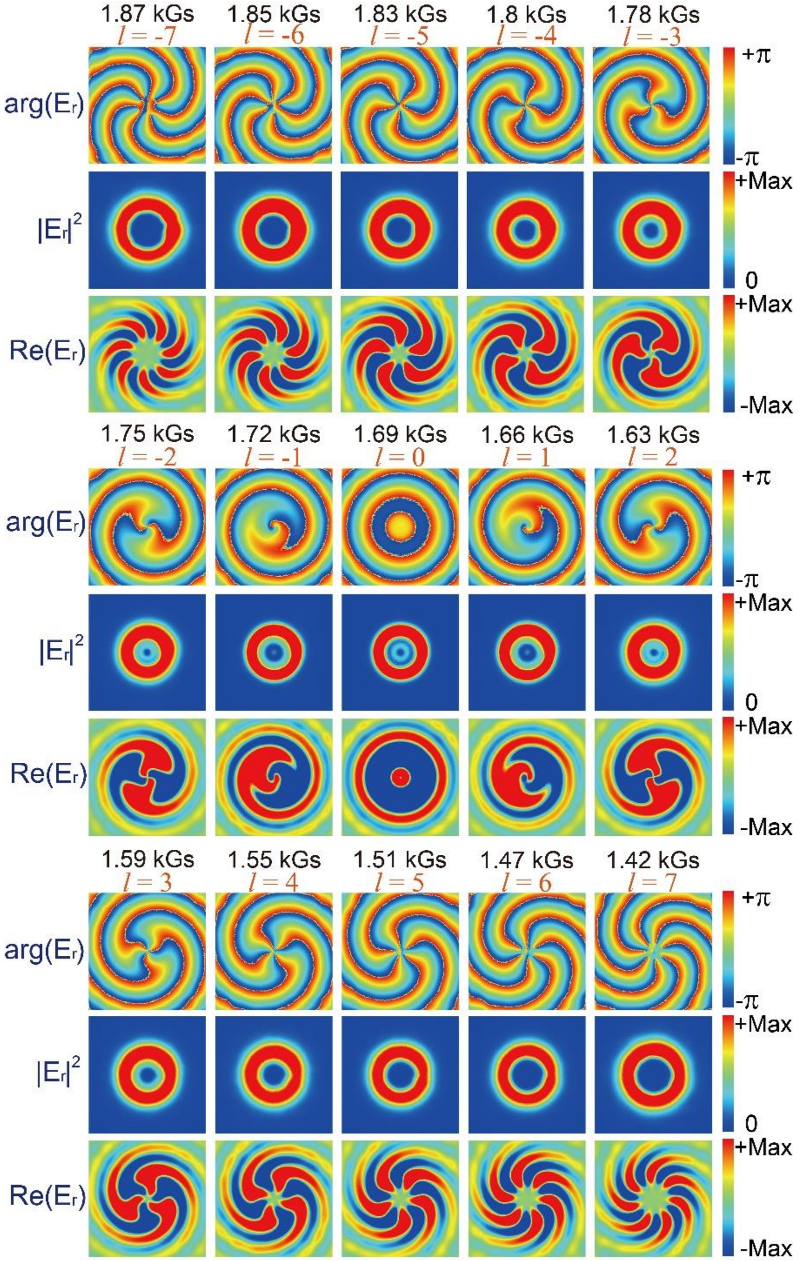

Fig. S9. OAM tuning through external magnetic field. Simulated phase, intensity and real part profiles of radial electric-field components of emitted OAM states for various external magnetic fields (H_0). The ring cavity is composed of 43 meta-cells with p = 6 mm and a = 4 mm. The frequency is fixed at f = 8.5 GHz. The resulting topological charge of the OAM radiation can be seen by the number of 2π phase evolution along the circumference, which ranges from –7 to +7 for the H_0 tune range [1.42, 1.87] kGs.

10. Tailoring the output OAMs through structural parameter

According to Fig. 6c, the OAM order of emitted vortex beam can also be tailored by varying structural parameter d. To verify this, Fig. S10 shows the emitted OAM states for different parameter d at $f = 8.5$ GHz and $H_0 = 1785$ Gs. The topological charge of the OAM state can be tuned from $l = -7$ to $+7$ by choosing different d values, which agrees well with our theoretical calculation shown in Fig. 6c.

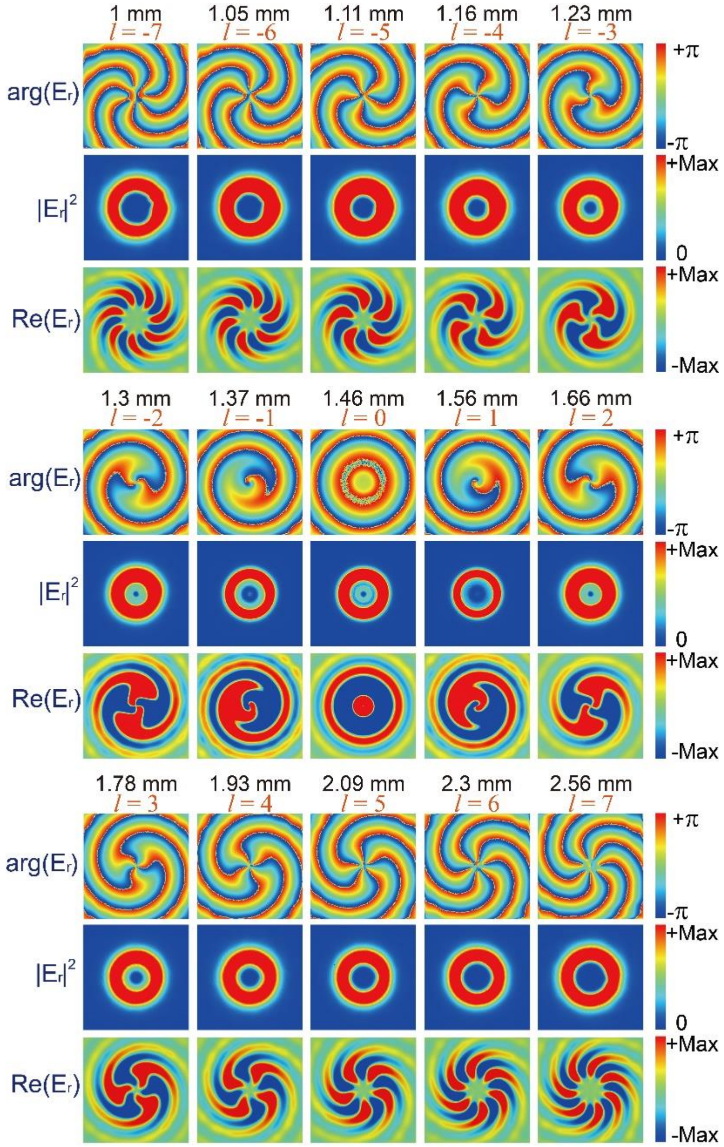

Fig. S10. OAM controlling by structural parameter. Simulated phase, intensity and real part profiles of radial electric-field components of emitted OAM states tuned

through the structural parameter d. The ring cavities are composed of 43 meta-cells with p = 6 mm and a = 4 mm. The frequency is fixed at f = 8.5 GHz and external magnetic field is H_0 = 1785 Gs. The resulting topological charge of the OAM radiation can be seen by the number of 2π phase evolution along the circumference, which ranges from −7 to +7 for the chosen d values, matching well with the results in Fig. 6c.

11. Tunable ring-cavity OAM source based on the USMP-driven metasurface using an alternative approach

A unidirectional ring cavity needs not exclusively rely on a radial magnetic field, it can also operate under a uniform external magnetic field. In microwave regime, such a ring cavity can be constructed using a unidirectional waveguide, which is a radially layered metal-air-YIG-metal structure. This waveguide terminates with a pair of metal slabs along the axial direction (z-axis) of the ring cavity. Under a uniform external magnetic field in the z direction, this ring structure can sustain resonant modes with unidirectionally circulating energy flows. Furthermore, by introducing periodic holes on the upper metal wall of the ring cavity, this setup is capable of generating OAM beams, offering similar capabilities to former unidirectional ring cavities (Fig. S11).

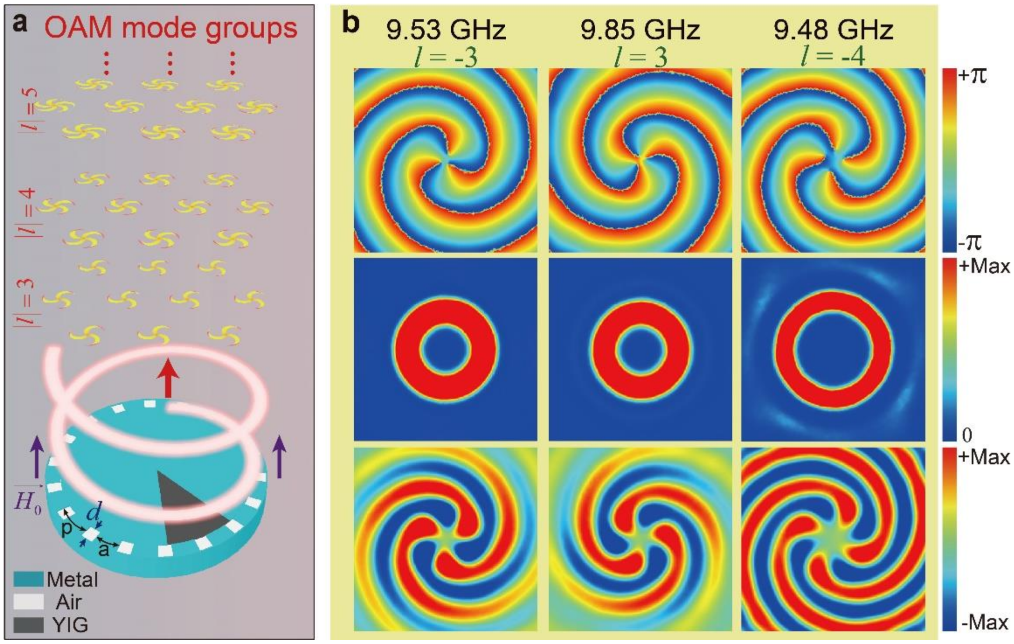

Fig. S11. Tunable ring-cavity OAM source based on the USMP-driven metasurface operating with a uniform external magnetic field. a Schematic of a ring-cavity OAM source. **b** Profiles of simulated far-field phase (top), intensity (middle) and real part (bottom) of the field component E_z of generated OAM beams at different frequencies. The geometric parameters of the ring cavity are as follows: the ring radius R = 41 mm, p = 6 mm, a = 5 mm, d = 1 mm. Here, H_0 = 1785 Gs, p = 6 mm, a = 5 mm, d =1 mm, and the diameter of the YIG ring is 82 mm. The external magnetic field points in the z-axis direction, with the amplitude H_0 = 1785 Gs.

12. Terahertz USMPs with unique dispersion

It should be entirely feasible to extend the concept of USMP-driven metasurfaces to the terahertz regime. The existence of USMPs closely relies on strong magneto-optical materials. In the terahertz regime, semiconductors (e.g., InSb) can exhibit strong magneto-optical effects under normal magnetic field strength. It was demonstrated both theoretically and experimentally that terahertz USMPs can be sustained by the interface between the magnetized semiconductor and opaque material within the upper bandgap of the magnetized semiconductor[1,2], which is topologically nontrivial. Such USMPs are robust against defects and even nonlocal effects. Our theoretical studies further revealed that in the upper bandgap of magnetized semiconductors, the semiconductor-dielectric-metal structure can support robust (terahertz) USMPs with lower propagation losses compared to the former[3]. However, all terahertz USMPs previously reported only have limited phase controllability, as their dispersion curves could only cover half of the light cone in air, thus failing to meet the requirements for constructing metasurfaces. Therefore, we investigate a new physical model for terahertz USMPs, namely the semiconductor-dielectric-opaque material-metal layered structure (Fig. S12a), and then find that the dispersion curve of USMP in this structure can cover the entire air light cone (Fig. S12b), thus possessing complete phase controllability.

In the presence of an external magnetic field ($\vec{B}_0 = \vec{y}B_0$), semiconductor can exhibit strong gyroelectric effect, with a relative permittivity tensor in the form of $\overleftrightarrow{\varepsilon} = [\varepsilon_1, 0, -i\varepsilon_2; 0, \varepsilon_3, 0; i\varepsilon_2, 0, \varepsilon_1]$ [3,4]. For the TM polarization, the dispersion relation of bulk modes in the magnetized semiconductor is $k = \sqrt{\varepsilon_v}(\omega/c)$, where $\varepsilon_v = \varepsilon_1 - \varepsilon_2^2/\varepsilon_1$. There exist two bandgaps ($\varepsilon_v < 0$) in the magnetized semiconductor, either below or above the plasma frequency (ω_p), and the upper one is completely created by the external magnetic field, thus possessing nontrivial topological property. In the semiconductor-dielectric-opaque material-metal layered structure, surface magnetoplasmons (SMPs)

exhibit TM-polarized field, and its dispersion relation can be derived analytically from Maxwell's equations, expressed as

$$\gamma - \frac{\varepsilon_2}{\varepsilon_1}k + \varepsilon_v \frac{\alpha_r \tanh(\alpha_r d_r)/\varepsilon_r + \alpha_m \tanh(\alpha_m d_m)/\varepsilon_m}{1 + (\varepsilon_r \alpha_m/\varepsilon_m \alpha_r)\tanh(\alpha_r d_r)\tanh(\alpha_m d_m)} = 0, \qquad \text{(S6)}$$

where k is the propagation constant, $\gamma = \sqrt{k^2 - \varepsilon_v k_0^2}$, $\alpha_m = \sqrt{k^2 - \varepsilon_m k_0^2}$, and$\alpha_r = \sqrt{k^2 - \varepsilon_r \mu_r k_0^2}$, ($k_0$ is the wavenumber in free space); ε_r (ε_m) and d_r (d_m) are the relative permittivity and thickness of the dielectric (opaque medium) layer. Here, the semiconductor is assumed to be InSb with $\omega_p = 4\pi \times 10^{12}$ rad/s. Figure S12b and c show the dispersion relation of USMP in the upper bandgap of the magnetized semiconductor. For the central frequency ($\omega = 1.1994\omega_p$) of the bandgap, the propagation constant can be tuned from $-k_0$ to k_0 (vacuum wave number) by varying the dielectric-layer thickness or external magnetic field, confirming that USMP possesses complete phase controllability.

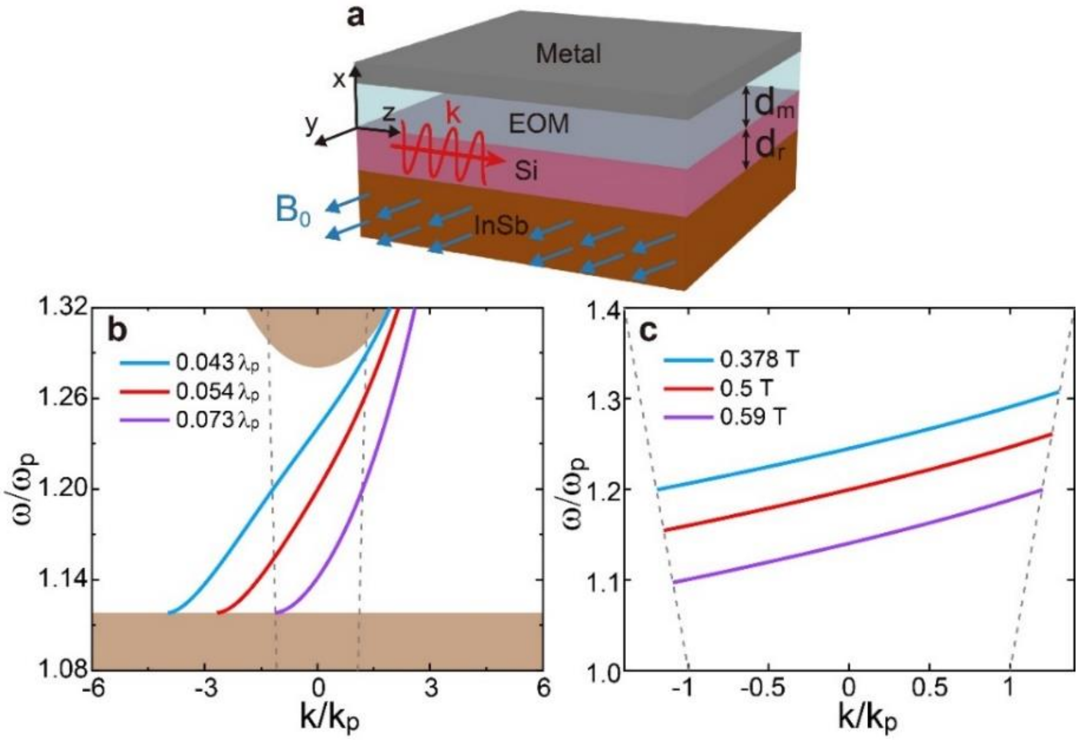

Fig. S12. Robust USMPs with complete phase controllability in terahertz regime. **a** Schematic of the plasmonic structure supporting robust USMP at terahertz frequencies. EOM: electrically opaque material. **b, c** Dispersion curves of USMPs in

the upper bandgap of the magnetized semiconductor for different d_r and B_0 values. The shaded areas represent the zones of bulk modes in the magnetized semiconductor, and the dashed lines represent light lines in free space. The external magnetic field in **b** is $B_0 = 0.5$ T, and the thickness of the dielectric layer in **c** is $d_r = 0.054\lambda_p$ (λ_p is the vacuum wavelength for ω_p). The other parameters of the guiding system are $\varepsilon_r = 11.68$, $\varepsilon_m = -7$, and $d_m = 0.1\ \lambda_p$.

13. Structural details of the designed USMP-driven metasurfaces for wavefront control

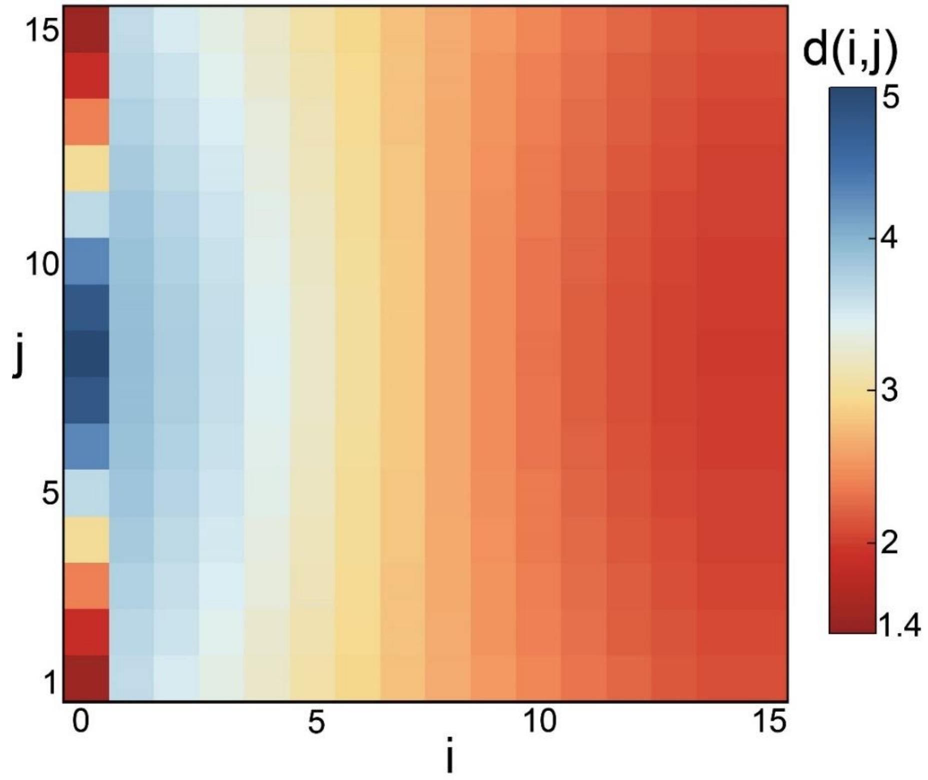

Fig. S13. Distributions of *d*(*i*, *j*) for the designed USMP-driven metasurface studied in Figure 3 of the main-text.

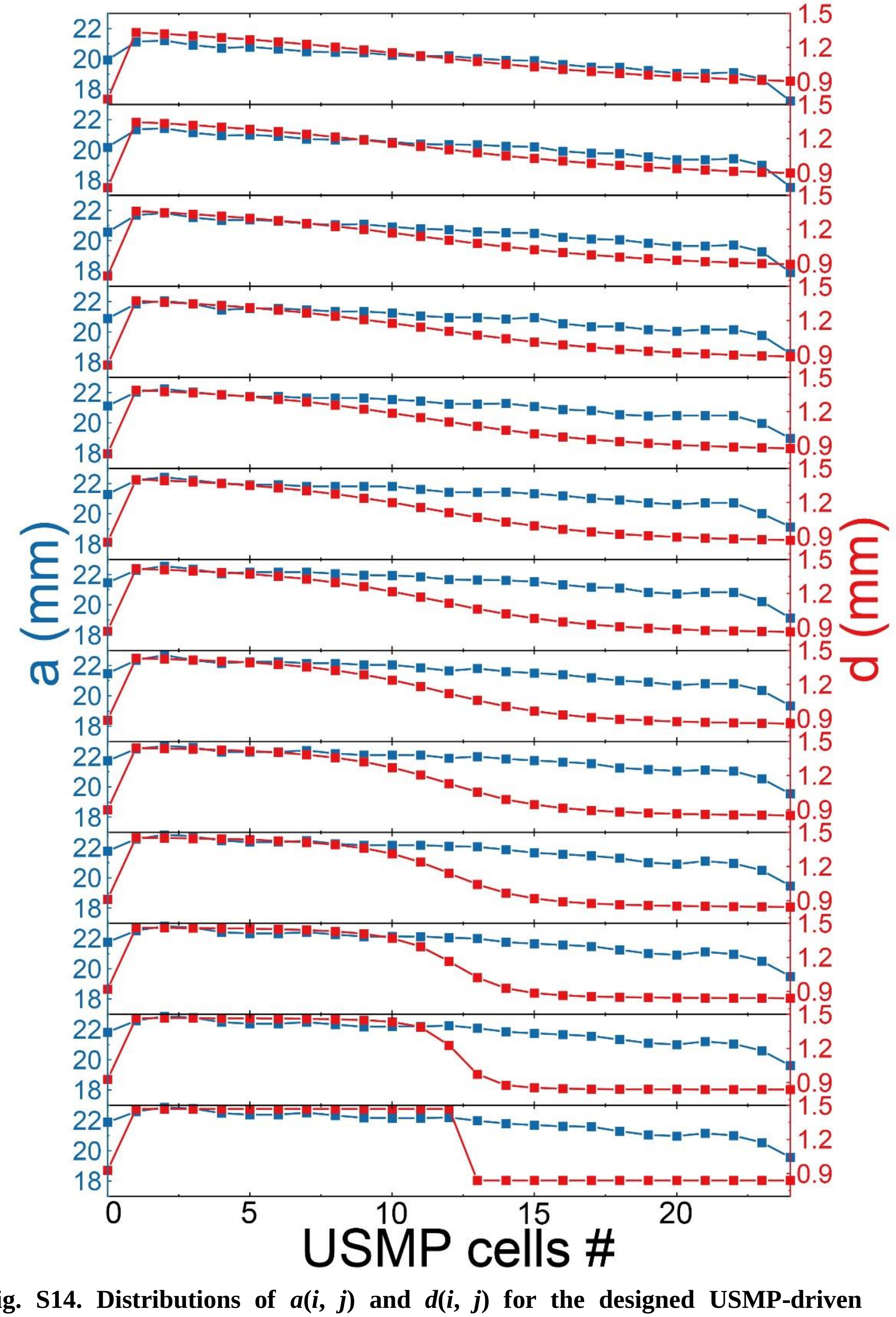

Fig. S14. Distributions of *a*(*i*, *j*) and *d*(*i*, *j*) for the designed USMP-driven metasurface studied in Figure 4 of the main-text. This USMP-driven metasurface is symmetric in structure, hence the remaining 12 lines of structural parameters exhibit a symmetric distribution with the preceding 12 lines.

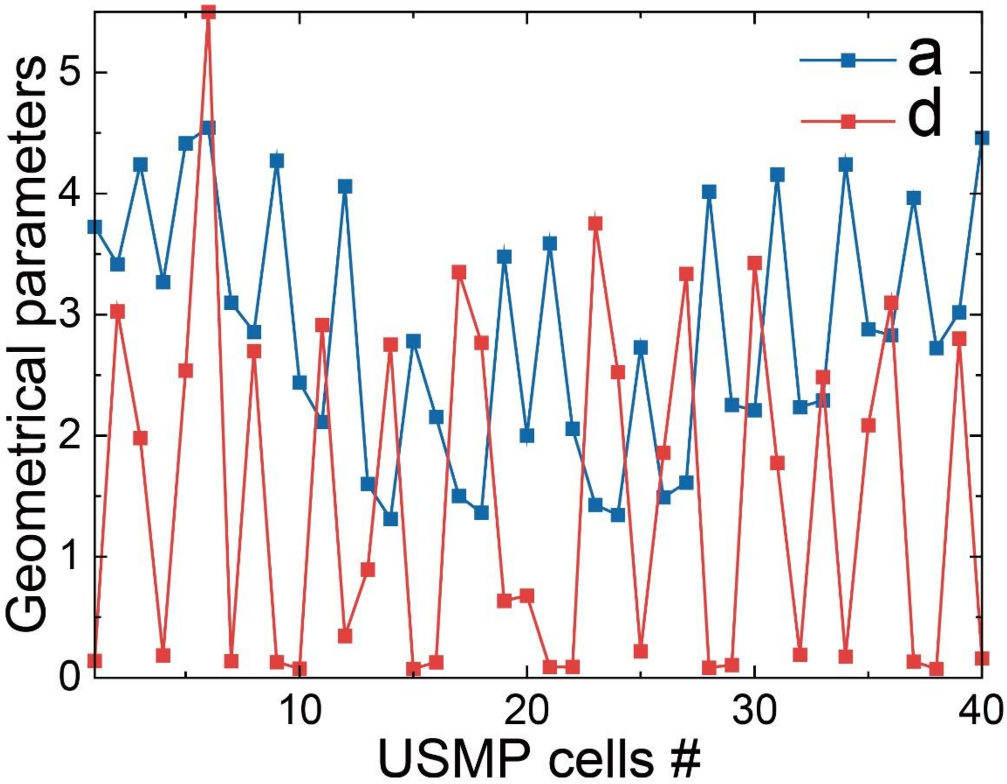

Fig. S15. Distributions of *a*(*i*, *j*) and *d*(*i*, *j*) for the designed USMP-driven metasurface studied in Figure 5 of the main-text.

14. Comparison of the proposed USMP-driven metasurfaces with selected prior art.

Representative Works	Major Principle	Capabilities and Key Advantages	Disadvantages
[5], [6]	Artificially designed meta-atoms impart abrupt phase shift to the incident wave from free space	complete manipulation of amplitude, phase, and polarization of light fields in both near-field and far-field regimes with an ultra-thin planar framework.	On-chip integration becomes difficult when driven by free-space waves. Controlling wavefronts at deep-subwavelength scales remains challenging.
[7]	Dressing metasurfaces onto reciprocal waveguide	Arbitrary far-field beam shaping by extracting light from a waveguide.	Necessitating meta-atoms with complex structures at subwavelength scales. Unable to manipulate wavefronts at deep-subwavelength scales.
[8], [9]	Single-layered leaky-wave metasurface supporting quasi-bound states in the continuum	Arbitrary control of far-field beam shaping, including polarization, with four degrees of freedom.	Manipulating wavefronts at subwavelength scales is beyond its capabilities.
This work	Directly extracting EM wave from unidirectional waveguide with unique dispersion	Complete and arbitrary ultra-precise control of near-field and far-field wavefront shapes at deep-subwavelength scales.	Arbitrary control of the polarization of wavefronts remains challenging.

Supplementary Table S1: Comparison of the proposed USMP-driven metasurface with selected prior art.

References

1. S. A. H. Gangaraj and F. Monticone, Do truly unidirectional surface plasmon-polaritons exist?. *Optica* **6**, 1158-1165 (2019).
2. Y. Liang, S. Pakniyat, Y. Xiang, Y. Xiang, J. Chen, F. Shi, G. W. Hanson, and C. Cen, Tunable unidirectional surface plasmon polaritons at the interface between gyrotropic and isotropic conductors. *Optica* **8**, 952-959 (2021).
3. J. H. Yan, Q. Shen, H. Zhang, S. Li, H. Tang, and L. F. Shen, Broadband unidirectional surface plasmon polaritons with low loss. *Opt. Express* **31**, 35313-35329 (2023).
4. J. J. Brion, R. F. Wallis, A. Hartstein, and E. Burstein, Theory of Surface Magnetoplasmons in Semiconductors. *Phys. Rev. Lett.* **28**, 1455-1459 (1972).
5. N. F. Yu, P. Genevet, M. A. Kats, F. Aieta, J.-P. Tetienne, F. Capasso, and Z. Gaburro, Light propagation with phase discontinuities: generalized laws of reflection and refraction. *Science* **334**, 333-337 (2011).
6. S. L. Sun, Q. He, S. Y. Xiao, Q. Xu, X. Li, and L. Zhou, Gradient-index meta-surfaces as a bridge linking propagating waves and surface waves. *Nat. Mater.* **11**, 426-431 (2012).
7. X. X. Guo, Y. M. Ding, X. Chen, Y. Duan, and X. J. Ni, Molding free-space light with guided wave-driven metasurfaces. *Sci. Adv.* **6**, eabb4142 (2020).
8. G. Y. Xu, A. Overvig, Y. Kasahara, E. Martini, S. Maci, and A. Alù, Arbitrary aperture synthesis with nonlocal leaky-wave metasurface antennas. *Nat. Commun.* **14**, 4380 (2023).
9. H. Q. Huang, A. C. Overvig, Y. Xu, S. C. Malek, C.-C. Tsai, A. Alù, and N. F. Yu, Leaky-wave metasurfaces for integrated photonics. *Nat. Nanotechnol.* **18**, 580-588 (2023).
